# Highly Pathogenic Avian Influenza A(H5N1) Virus Stability in Irradiated Raw Milk and Wastewater and on Surfaces, United States

**DOI:** 10.3201/eid3104.241615

**Published:** 2025-04

**Authors:** Franziska Kaiser, Santiago Cardenas, Kwe Claude Yinda, Reshma K. Mukesh, Missiani Ochwoto, Shane Gallogly, Arthur Wickenhagen, Kyle Bibby, Emmie de Wit, Dylan Morris, James O. Lloyd-Smith, Vincent J. Munster

**Affiliations:** National Institute of Allergy and Infectious Diseases, Hamilton, Montana, USA (F. Kaiser, K.C. Yinda, R.K. Mukesh, M. Ochwoto, S. Gallogly, A. Wickenhagen, E. de Wit, V.J. Munster); University of California, Los Angeles, California, USA (S. Cardenas, D. Morris, J.O. Lloyd-Smith); University of Notre Dame, Notre Dame, Indiana, USA (K. Bibby)

**Keywords:** influenza, viruses, food safety, respiratory infections, H5N1, milk, wastewater, stability, half-life, United States

## Abstract

We measured stability of infectious influenza A(H5N1) virus in irradiated raw milk and wastewater and on surfaces. We found a relatively slow decay in milk, indicating that contaminated milk and fomites pose transmission risks. Although the risk is low, our results call for caution in milk handling and disposal from infected cattle.

Since its first detection in cattle in March 2024, highly pathogenic avian influenza (HPAI) A(H5N1) virus has caused a multistate outbreak in dairy cows in the United States (*1*). The Eurasian lineage goose/Guangdong H5 clade 2.3.4.4b virus has been endemic in the United States since winter 2021–22, causing the outbreak in dairy cows (*2*,*3*), and has caused a series of epizootic outbreaks in mammal species in the United States. (*3*,*4*). During the ongoing US dairy cow epidemic, zoonotic and cross-species spillovers of H5N1 to farm workers and other mammalian and avian species near dairy farms have occurred (*5*–*7*).

H5N1 virus replicates in mammary gland epithelial cells of dairy cows. Replication inside the udder results in high viral titers of up to 10^8.8^ 50% tissue culture infectious dose per milliliter in milk from infected cows (*8*,*9*). Key drivers of cow-to-cow, zoonotic, and cross-species transmission might be through direct, environmental (through contaminated milk or wastewater streams), or surface (contaminated milking equipment) contact (*10*,*11*). To examine environmental and mechanical H5N1 virus transmission, we evaluated the stability of infectious H5N1 virus in irradiated raw milk and wastewater and on surfaces.

## The Study

We assessed the decay rates and corresponding half-lives of H5N1 virus in irradiated raw milk; on polypropylene, stainless steel, and rubber surfaces; and in irradiated wastewater from a treatment plant. We performed irradiation of raw milk and wastewater to avoid bacterial outgrowth that would prevent further downstream analyses because of cytotoxicity.

We spiked fresh, raw, gamma-irradiated cow milk and wastewater with HPAI H5N1 clade 2.3.4.4b virus isolated from the ongoing US dairy cattle outbreak (strain A/bovine/OH/B24OSU-342/2024). We tested samples of spiked fluids daily for 7 days. We deposited four 12.5-µL drops of spiked irradiated raw milk on stainless steel, polypropylene, and nitrile rubber disks to evaluate surface stability. We collected daily samples by rinsing the disks with minimal essential media tissue culture media and performed all experiments in triplicate by using biologic replicates and quantifying endpoint titration on MDCK cells (Appendix).

We evaluated the stability of H5N1 virus in irradiated raw milk and on surfaces at 4°C and 22°C and in wastewater at 22°C. We inferred posterior distributions for virus decay rates and half-lives by using a Bayesian regression model. We report inferred values as the posterior median (95% credible interval [CrI]).

**Figure 1:**
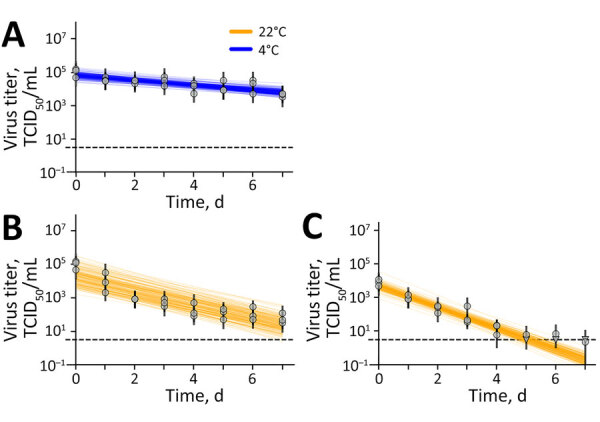
Results of experimental testing of highly pathogenic avian influenza A(H5N1) virus stability in irradiated raw milk and wastewater, United States. A, B) Virus stability in irradiated raw milk at (A) 4°C and (B) 22°C. C) Virus stability in irradiated wastewater at 22°C (orange). Vertical lines represent random draws from the joint posterior distribution of the exponential decay rate and the initial virus titer, where the intercept of each line is the initial titer and the slope is the negative of the decay rate. Dashed horizontal lines show 10^0.5^ TCID_50_/mL of medium and represent the approximate detection limit. Individual data points are represented as circles (above limit of detection) or triangles (below limit of detection). TCID_50_, 50% tissue culture infectious dose.

In irradiated raw milk, we measured a virus half-life of 2.1 (95% CrI 1.5–3.4) days at 4°C and 0.74 days (95% CrI 0.60–0.96) at 22°C (Figure 1). The time needed for a 10 log_10_ reduction in virus titer was 69 (95% CrI 51–112) days at 4°C and 24 (95% CrI 20–32) days at 22°C. Those results underline the potential of H5N1 virus to stay infectious in milk for multiple weeks, especially if the milk is refrigerated, if the initial titer is sufficiently high.

**Figure 2:**
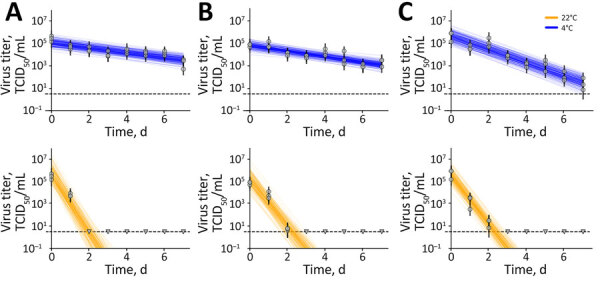
Results of experimental testing of highly pathogenic avian influenza A(H5N1) virus stability on surfaces, United States. Surface stability of infectious H5N1 in milk at 4°C (blue) and 22°C (orange) was tested on polypropylene (A), stainless steel (B), and nitrile rubber material (C). Vertical lines represent random draws from the joint posterior distribution of the exponential decay rate and the initial virus titer, where the intercept of each line is the initial titer and the slope is the negative of the decay rate. The dashed horizontal lines are at 10^0.5^ TCID_50_/mL of medium and represent the approximate limit of detection. Individual data points are represented as circles (above limit of detection) or triangles (below limit of detection). TCID_50_, 50% tissue culture infectious dose.

We studied surface stability of H5N1 virus on stainless steel, polypropylene, and rubber surfaces at 4°C with 80% relative humidity and at 22°C with 65% relative humidity. At 4°C, we measured a half-life of 1.4 (95% CrI 1.1–2.1) days on polypropylene, 1.2 (95% CrI 1.0–1.7) days on stainless steel, and 0.51 (95% CrI 0.45–0.59) days on rubber. At 22°C, we measured half-life values of 0.11 (95% CrI 0.08–0.14) days (2.5 [95% CrI 1.6–3.4] hours) on polypropylene, 0.14 (95% CrI 0.11–0.18) days (3.3 [95% CrI 2.5–4.3] hours) on stainless steel, and 0.14 (95% CrI 0.12–0.16) days (3.31 [95% CrI 2.86–3.87] hours) on rubber (Figure 2). Decay rates of infectious H5N1 virus were comparable on stainless steel and polypropylene surfaces but were ≈10 times faster at 22°C (room temperature) than at 4°C. Stability on rubber material was no different at 22°C but lower at 4°C compared with polypropylene or stainless steel. A 10 log_10_ reduction in infectious virus titer was achieved at 4°C after 45 (95% CrI 35–62) days on polypropylene, after 40 (95% CrI 32–57) days on steel, and after 16.95 (95% CrI 15.07–19.64) days on rubber. In comparison, at 22°C, the 10 log_10_ decrease took 3.6 (95% CrI 2.7–4.6) days on polypropylene, 4.7 (95% CrI 3.7–5.9) days on stainless steel, and 4.59 (95% CrI 3.96–5.36) days on rubber.

**Figure 3:**
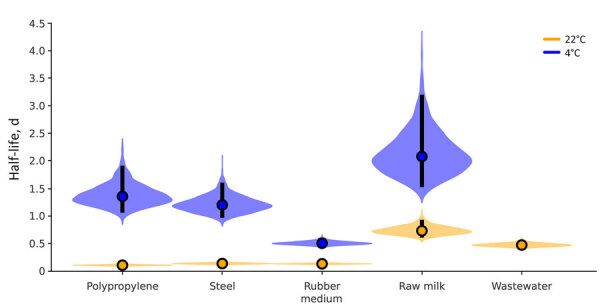
Violin plots showing results of experimental testing of highly pathogenic avian influenza A(H5N1) virus stability in irradiated raw milk and wastewater and on surfaces, United States. Plots show the posterior distribution of the half-life of viable virus at each condition, determined from the estimated decay rates. Viral decay was calculated for H5N1 virus in irradiated raw milk at 22°C and 4°C, in irradiated wastewater at 22°C, and on polypropylene, steel, and rubber surfaces at 22°C and 4°C. The point at the center of each violin is the posterior median estimate, and the vertical black bars show 95% credible intervals (2.5%–97.5%).

In wastewater at 22°C, the half-life of infectious H5N1 virus was 0.48 (95% CrI 0.42–0.56) days (Figure 3). A 10 log_10_ reduction of infectious virus in wastewater took 16 (95% CrI 14–19) days if stored at 22°C.

## Conclusions

We investigated the environmental persistence of infectious H5N1 virus in irradiated milk, on contaminated surfaces, and in irradiated wastewater. For irradiated raw milk and surface stability, we tested refrigerator (4°C) and room temperature (22°C) settings. H5N1 virus showed high stability and a monophasic, exponential decay pattern in all experiments. Refrigeration led to slower virus inactivation (*12*). Inactivation of the virus was slower in milk than in wastewater, possibly because of stabilization arising from milk’s high protein content, similar to mpox virus, where increasing protein content has been reported to improve stability (*13*). However, previous experiments were conducted in virus-spiked irradiated milk and wastewater (*13*), and the effect of microorganisms was not investigated.

Based on the estimated inactivation rate and high virus titer of 10^8^ 50% tissue culture infectious dose per milliliter (detected during the current outbreak) (*8*), detectable quantities of infectious virus could theoretically persist in refrigerated irradiated raw milk for 45 (95% CrI 33–73) days. On surfaces, we did not find substantial differences in virus stability among exposure to stainless steel, polypropylene, or rubber at 22°C but detected a high dependency on temperature. Another study comparing H5N1 virus surface stability on stainless steel and rubber from milking equipment found comparable virus stability between the 2 surfaces, underlining the potential risk for fomite transmission (*10*). The half-life of 0.48 (95% CrI 0.42–0.56) days (12 [95% CrI 10–13] hours) in wastewater shows persistence on a time scale that may lead to exposure of humans or animals when in contact with contaminated wastewater or surface water.

The relatively high stability of H5N1 virus we report, combined with reports of high H5N1 virus titers in milk from infected cows, highlights the potential for virus transmission by contaminated milk or fomites. That high stability is consistent with postulated cow-to-cow transmission during the milking process and exposure to infected cattle herds, leading to infections in dairy workers and other animal species at affected dairy farms. Furthermore, the high virus titers in milk pose a serious risk for transmission to a poultry industry that neighbors dairy farms or dairy processing sites. Previous studies on avian H5N1 and H1N1 viruses reported the same prolonged environmental stability described here for bovine HPAI H5N1 virus (*10*,*14*). In addition, although wastewater transmission risk appears to remain low, detection of H5N1 sequences in wastewater during weekly wastewater sampling in 10 urban areas throughout Texas suggested widespread H5N1 genetic material in wastewater in states affected by the outbreak in dairy cattle (*15*).

In conclusion, our results indicate low H5N1 virus infection risk through wastewater but higher potential risk from exposure to contaminated milk. Research into transmission risk through different pathways and the probability of infection arising from different doses and routes of exposure to H5N1 is ongoing, but our results call for particular caution in handling and disposing of milk from infected cattle.

This article was preprinted at https://www.biorxiv.org/content/10.1101/2024.10.22.619662v1.

Appendix Additional information for highly pathogenic avian influenza A(H5N1) virus stability in irradiated raw milk and wastewater and on surfaces, United States.
