# Supplementary material for: Highly Pathogenic Avian Influenza A(H5N1) Virus Stability in Irradiated Raw Milk and Wastewater and on Surfaces, United States
Source: Emerg Infect Dis. 2025 Apr;31(4):833–7. doi: 10.3201/eid3104.241615 (PMC11950256; doi:10.3201/eid3104.241615)
Supplement: Appendix — Additional information for highly pathogenic avian influenza A(H5N1) virus stability in irradiated raw milk and wastewater and on surfaces, United States. [file 24-1615-Techapp-s1.pdf]

Article DOI: https://doi.org/10.3201/eid3104.241615

EID cannot ensure accessibility for supplementary materials supplied by authors. Readers who have difficulty accessing supplementary content should contact the authors for assistance.

Highly Pathogenic Avian Influenza A(H5N1) Virus Stability in Irradiated Raw Milk and Wastewater and on Surfaces, United States

Appendix

Experimental Methods

Biosafety and Ethics

This study was approved by the Institutional Biosafety Committee (IBC) and performed in high biocontainment (BSL4) at Rocky Mountain Laboratories (RML), National Institute of Allergies and Infectious Diseases, National Institutes of Health. All sample processing and sample removal from high containment followed IBC-approved standard operating protocols (SOPs).

Viruses and Cells

The HPAI H5N1 strain A/bovine/OH/B24OSU-342/2024 was grown and titrated on MDCK cells in minimal essential media (MEM) (Sigma-Aldrich, St. Louis, MO, USA) containing 4 µg/mL trypsin, 2 mM L-glutamine, 50 U/mL penicillin, 50 µg/mL streptomycin, 1% nonessential amino acids (NEAA), 20 mM HEPES (all Thermo Fisher Scientific, Waltham, MA, USA). MDCK cells were maintained in MEM supplemented with 10% fetal bovine serum (FBS) (Wisent Inc., St. Bruno, Canada), 2 mM L-glutamine, 50 U/mL penicillin, 50 µg/mL streptomycin, 1% NEAA, and 20 mM HEPES.

Viral RNA Detection

Quantitative reverse transcription-PCR was performed on RNA samples extracted from swabs by using QiaAmp Viral RNA or tissues by using RNeasy kits (Qiagen, Germantown, MD, USA). Viral RNA was detected with a Luna 1-step RT-qPCR kit (New England BioLabs) designed to amplify total viral RNA from the matrix protein. Primers and probe used to amplify

the viral RNA were as follows: forward primer 5′-AAGACCAATCCTGTCACCTCTGA-3′; reverse primer 5′-CAAAGCGTCTACGCTGCAGTCC-3′; probe FAM-TTTGTGTTCACGCTCACCGTGCC-TAMRA. Dilutions of RNA standards were numerically determined by droplet digital PCR, run in parallel, and used to estimate viral RNA genome copy numbers.

Virus Titration

Viruses were titrated by endpoint dilution in MDCK cells. MDCK cells were inoculated with 10-fold serial dilutions of the environmental stability samples. One hour after inoculation, cells were washed twice with phosphate-buffered saline (PBS) and supplemented with infection medium (MEM supplemented with 4 µg/mL L-1-tosylamido-2-phenylethyl chloromethyl ketone [TPCK] trypsin, 2 mM L-glutamine, 50 U/mL penicillin, 50 µg/mL streptomycin, 1% NEAA, and 20 mM HEPES). Three days after inoculation, the supernatants of infected cell cultures were tested for agglutination activity by using turkey red blood cells as an indicator of infection of the cells.

Experimental Design for Stability Studies

For wastewater collection, ≈1 L of untreated primary influent was collected from a municipal wastewater treatment plant in northern Indiana, USA, receiving an average flow of 11 million gallons per day (MGD). Immediately after being collected, the sample was stored overnight at −80°C and shipped to the Rocky Mountain Laboratories (RML) overnight on ice. The sample was then stored at −80°C at RML until the experiments described herein were begun (*1*). Raw cow milk was collected from a local dairy farm in Montana. The milk had a fat content of 4.98% and 3.51% protein (CentralStar Laboratories). Before the experiment, fresh, raw cow milk was irradiated with 2 Mrad and wastewater at 8 Mrad to ensure inactivation of potential bacterial contaminants. Milk and wastewater were spiked with 10^6 50% tissue culture infectious dose ($TCID_{50}$)/mL highly pathogenic avian influenza virus H5N1 strain A/bovine/OH/B24OSU-342/2024. The virus-matrix suspension was then aliquoted into 2-mL closed tubes (2-mL polypropylene vials; Sarstedt) and stored at the respective condition (4°C or 22°C). To maintain the desired temperatures consistently, we kept the aliquots in an environmental chamber (MMM Group, https://www.mmm-medcenter.com) at 22°C and 65% relative humidity and a refrigerator (Isotemp Laboratory Refrigerator; Fisherbrand) at 4°C and 80% relative humidity. Samples were

collected on day 0, 1, 2, 3, 4, 5, 6, and 7 timepoints and stored at −80°C until time of titration. All experiments were performed in triplicate.

Experimental Design for Virus Stability on Surfaces

Surface stability was evaluated on plastic (polypropylene; ePlastics), AISI 304 alloy stainless steel (Metal Remnants), and rubber material, chosen to represent plastic, metal, and synthetic nitrile rubber (McMaster-Carr) surfaces common in the dairy farming and industry. A 50-μL H5N1-milk suspension (2.5×10^7 $TCID_{50}$/mL; 1.25×10^6 per 50 μL) was deposited in 4 drops (12.5 μL each) on polypropylene, stainless steel, or nitrile rubber disks. Disks were placed on plastic plates and contained in HEPA-filtered boxes under controlled environmental conditions. Experiments were carried out at 22°C and 65% humidity and 4°C and 80% humidity in an environmental chamber or laboratory refrigerator. Deposited virus was recovered by rinsing with 1 mL MEM infection media at predefined timepoints (0, 1, 2, 3, 4, 5, 6, and 7 days), and samples were stored at −80°C until time of titration. All experiments were performed in triplicate.

Statistical Analyses

The statistical analyses closely follow those in Kaiser et al. (*2*). As in that manuscript, we used a Bayesian approach to infer sample titers and virus half-lives from raw endpoint titration well data. We used Numpyro (https://num.pyro.ai/en/stable/getting_started.html) and Python 3 (https://www.python.org) to specify and fit models. The principal difference between the analysis here and that in Kaiser et al. (*2*) is the inclusion of an additional Normal error term for predicted titers during environmental decay.

As previously described, we model well positivity in titration data with a Poisson single hit model; the hit probability depends on the initial virion concentration and the per-virion cell infection probability. In other words, if susceptible cells in a well are inoculated with infectious virions, the number of virions that infect cells and replicate is Poisson distributed. However, only 1 virion needs to successfully enter a cell and replicate to produce a positive infection result for the well, so we model the well positivity as the probability that the Poisson random variable is greater than zero. The mean is measured in units of 50% tissue infectious dose ($TCID_{50}$), so we do not need to consider the per-virion cell infection probability. We model the decay of viable virus as a linear decay in $\log_{10}$ units (*2*); however, to account for possible overdispersion in

sample collection, we add an error term. For each sample k in experimental condition j, the quantity of viable virus at the time of sampling $v_{kj}(t_{kj})$ is given by

$$v_{kj}(t_{kj}) = v_{kj}(0) - l_j t_{jk} + \epsilon_{kj},$$

where $v_{kj}(t_{kj})$ is measured in $\log_{10}$ $TCID_{50}$, l_j is the rate of decay under condition j, and ϵ_{kj} is a Normally distributed error term with mean 0 and an inferred condition-specific standard deviation σ_j. The initial virus concentration $v_{kj}(0)$ is Normally distributed with inferred experimental condition-specific means $< v_0 >_j$ and standard deviations τ_j:

$$v_{kj}(0) \sim Normal(< v_0 >_j, \tau_j).$$

With that hierarchical approach, we model errors in the initial viral titer separately from errors in the collection and measurement of the titers during the decay process.

Prior Distributions

Here, Normal distributions are parameterized as Normal (mean, standard deviation), and positive truncated Normal distributions (Normal distributions truncated to have only positive support) are parameterized as PosNormal (mode, standard deviation), where the mode and standard deviation are those of the underlying Normal distribution.

We used the following prior distributions.

For the titers v_i, we used a weakly informative Normal prior in units of $\log_{10}$ $TCID_{50}$ per mL:

$$v_i \sim Normal(0, 10).$$

For the overdispersion of the titers, we placed a weakly informative PosNormal prior on the standard deviation of the error term ϵ_{kj}:

$$\sigma_j \sim PosNormal(0, 0.5).$$

For the experiment-specific mean initial $\log_{10}$ titers $< v_0 >_j$, we used a weakly informative Normal prior based on the target stock titer:

$$< v_0 >_j \sim Normal(5, 4).$$

For the standard deviation of the sample initial titers τ_j, we used a weakly informative positive truncated Normal distribution:

$\tau_j \sim PosNormal(0, 0.25)$.

For inferring the half-life of infectious virus in days, h_j, we placed a weakly informative Normal prior distribution on the natural log of the half-life:

$ln(h_j) \sim Normal(ln\ (1), ln\ (20))$.

References

1. Bivins A, Greaves J, Fischer R, Yinda KC, Ahmed W, Kitajima M, et al. Persistence of SARS-CoV-2 in water and wastewater. Environ Sci Technol Lett. 2020;7:937–42. PubMed https://doi.org/10.1021/acs.estlett.0c00730

2. Kaiser F, Morris DH, Wickenhagen A, Mukesh R, Gallogly S, Yinda KC, et al. Inactivation of avian influenza A(H5N1) virus in raw milk at 63°C and 72°C. N Engl J Med. 2024;391:90–2. PubMed https://doi.org/10.1056/NEJMc2405488
